# A new species of *Sinarella* Bryk, 1949 (Lepidoptera, Erebidae, Herminiinae) from Jiangxi, China

**DOI:** 10.3897/BDJ.13.e139845

**Published:** 2025-01-06

**Authors:** Jun Wu, Wen-Yu Liu, Hui-Lin Han

**Affiliations:** 1 School of Forestry, Northeast Forestry University, Harbin, China School of Forestry, Northeast Forestry University Harbin China; 2 Northeast Asia Biodiversity Research Center, Northeast Forestry University, Harbin, China Northeast Asia Biodiversity Research Center, Northeast Forestry University Harbin China; 3 Jinggangshan National Nature Reserve Administration of Jiangxi, Ji'an, China Jinggangshan National Nature Reserve Administration of Jiangxi Ji'an China; 4 Ministry of Education, Key Laboratory of Sustainable Forest Ecosystem Management, Northeast Forestry University, Harbin, China Ministry of Education, Key Laboratory of Sustainable Forest Ecosystem Management, Northeast Forestry University Harbin China

**Keywords:** China, Herminiinae, morphology, Noctuoidea, taxonomy

## Abstract

**Background:**

*Sinarella* Bryk, 1949 (Erebidae, Herminiinae) is a medium-sized, frail-bodied genus of moths that externally resembles other genera in the *Zanclognatha* generic complex. All known larvae of this genus feed on fresh leaves of moss. This genus is widely distributed from the Himalayas to Southeast Asia, Japan, Borneo, Sulawesi and New Guinea.

**New information:**

A new species of the genus *Sinarella* Bryk, 1949, *S.jinggangshana* **sp. nov.**, is described from Jinggangshan National Nature Reserve, Jiangxi Province, China. This species is morphologically similar to *S.takasago* Wu, Fu & Owada, 2013, *S.cristulalis* (Staudinger, 1892), *S.formosensis* Wu, Fu & Owada, 2013 and *S.japonica* (Butler, 1881), but differs in both external and male genitalia characters. Images of the male adults and their genitalia are provided.

## Introduction

The genus *Sinarella* was erected by Bryk (1949) with *S.stigmatophora* Bryk, 1949 (Type locality: North Korea) as the type species, initially including only the type and another, now synonymised, species: *S.aegrota* (Butler, 1879). However, the type species was later considered a junior synonym of *S.aegrota*. The genus is widely distributed in Asia: Owada (1987) and Owada (1992) revised the subfamily Herminiinae from Japan, describing eight *Sinarella* species, including two new species, *S.itoi* Owada, 1987 and *S.c-album* Owada, 1992 and designated a lectotype for *S.nigrisigna* (Leech, 1900) (Leech 1900, Owada 1987, Owada 1992). Sohn et al. (2004) documented *S.cristulalis* (Staudinger, 1892) (Staudinger 1892) in Korea and designated its lectotype. Subsequently, Kononenko and Han (2007) recorded six species distributed in Korea. Kononenko & Pinratana (2005, 2013) recorded *S.discisigna* (Moore, 1883), *S.griseola* Holloway, 2008 and *S.interrupta* (Wileman, 1915) in Thailand in the book series *The Moths of Thailand* (Moore 1883, Wileman 1915, Kononenko and Pinratana 2005, Holloway 2008, Kononenko and Pinratana 2013). Fu et al. (2013) considered that “Sinarella?interrupta” reported by Kononenko & Pinratana (2005) was a misidentification; it is actually *Mixomeliaformosensis* Hampson, recorded by Wang (1994). Since *M.formosensis* was an invalid manuscript name, it was subsequently formally renamed *S.formosensis* Wu, Fu & Owada, 2013. Fu et al. (2021) later transferred *S.interrupta* to the genus *Mesoplectra* Butler, 1879 (Butler 1879), based on characters of the male antennae, forelegs and female genitalia. Anikin et al. (2016) recorded five *Sinarella* species from the Russian Far East. Sondhi et al. (2021) suggested that *S.lunifera* (Moore, [1885]) (Moore 1885), from Sri Lanka, previously considered synonymous with *S.discisigna* by Beccaloni et al. (2003), may be a distinct species, with populations in the Western Ghats, potentially belonging to *S.lunifera* as well. In mainland China, Han et al. (2006) recorded three *Sinarella* moths in Jilin Province for the first time: *S.japonica* (Butler, 1881) (Butler 1881), *S.punctalis* (Herz, 1904) (Herz 1904) and *S.cristulalis*. Han et al. (2020)summarised that the genus now includes ten valid species, with five distributed across three provinces in northeast China.

Holloway (2008) divided *Sinarella* into two lineages: the type lineage includes species distributed in the temperate and subtropical regions of East Asia and another with an ash-grey color pattern, though this division requires further research for validation. Wu (2014) systematically reviewed *Sinarella* species from the Palearctic and Oriental Regions in his doctoral thesis, summarising the presence of 12 species in the Oriental Region, reporting 21 species in total, including 18 new species. He emphasised that there are still many species from Asia that remain undescribed.

Herein we described a new species, *Sinarellajinggangshana* sp. nov., from Jiangxi Province, China, based on morphological evidence. A species diagnosis is presented, along with illustrations of the adult and male genitalia and we provide a new diagnosis for the genus.

## Materials and methods

The type series was collected with a 220V/450W mercury vapour light and a DC black light in Jinggangshan National Nature Reserve, Jiangxi Province, China. Wingspan was measured from forewing apex-apex and the forewing length from the wing base to the apex. Standard methods for dissection and preparation of the genitalia slides were used following Kononenko and Han (2007). The specimens were photographed using a Nikon D700 camera, while the photographs of the genitalia slides were captured using an Olympus photo microscope aided by Helicon Focus software and then further processed using Adobe Photoshop CS6. The morphological terminology follows Kononenko and Han (2007).

All the type materials of the new species are deposited in the collection of the Northeast Forestry University (NEFU), Harbin, China.

Abbreviations used:

- NEFU, Northeast Forestry University, Harbin, China;
- HT, Holotype;
- PT, Paratype.

## Taxon treatments

### Sinarella

Bryk, 1949

695BCE72-3909-51B9-B03E-89232718372B

Sinarella Bryk, 1949 - Bryk (1949): 144.
Sinarella stigmatophora Bryk, 1949Bryk 1949

#### Diagnosis

The genus *Sinarella* Bryk, 1949 is characterised by long labial palpi, with the third segment slightly shorter than the second, with the terminal scales forming a small brush-like structure; the antennae are filiform. The outer margin of the forewing is rounded, with distinct spots and lines on dorsal surfaces, areole absent and the reniform spot is distinct. In the male genitalia, the uncus is stout with a hooked apex and the valva sclerotised, variable in shape, costa and saccular process slightly protruded, distal portion usually strongly acute, bifurcate or trifurcate; the vinculum is broad, “U” or “V” shaped; the aedeagus is typically curved at the mid-point, with the vesica usually bearing cornuti in variable number and shapes. In the female genitalia, the ductus bursae has a pair of longitudinal sclerites and varies in length; the corpus bursae is sac-shaped, longer than the ductus bursae and covered in scobination consisting of short spines of various shapes.

### Sinarella jinggangshana sp. nov.

77B6DDF2-C5A6-5873-9DAB-1FA2C7E83897

07EB1C85-7B99-4494-8BB8-FAF14125DE64

#### Materials

- **Type status:** Holotype. **Occurrence:** recordedBy: Hui-Lin Han, Jun Wu; sex: male; lifeStage: adult; disposition: in NEFU; occurrenceID: A7C2447F-812C-541F-B61D-C1025C0B9CF1; **Location:** country: China; stateProvince: Jiangxi; county: Ji'an; locality: Jinggangshan National Nature Reserve, Liujiaping Village; **Event:** eventDate: 26–29 Aug 2023
- **Type status:** Paratype. **Occurrence:** recordedBy: Hui-Lin Han; individualCount: 1; sex: male; lifeStage: adult; disposition: in NEFU; occurrenceID: FFF89B67-4F27-5AFE-BD55-99E3BAE459D1; **Location:** country: China; stateProvince: Jiangxi; county: Ji'an; locality: Jinggangshan National Nature Reserve, Liujiaping Village; **Event:** eventDate: 3–9 Aug 2021

#### Description

**Male** (Fig. 1a, b). Forewing length 9.5 mm, wingspan 20 mm in male. Head grey-white. Male antennae filiform, with a pair of long bristles on each segment. Labial palpus elongated, extending forwards, grey-white with black scales, third segment mostly black. Thorax and abdomen grey-white, sparsely mixed with black scales. Forewings broad, predominantly grey-white with a tinge of ochre, mixed with black scales; terminal area and fringe grey-brown; basal line black, only obvious towards costal margin; antemedial line sinuous, excurved; postmedial line black, dentate, prominently excurved in middle, with a faint ochreous shade along inner edge in posterior half; subterminal line nearly straight, dentate, inconspicuous, but forming 1–2 distinct black spots near R and R veins; terminal line consisting of elongate black dots. Orbicular spot barely discernible; reniform spot conspicuous, black with a grey-white mark in it, circular, very large. Fringe grey-brown, black-tipped. Hind-wings pale grey-white, with a dark grey-brown marginal shade; discal spot indistinct; median line dark brown, dentate, slightly excurved; subterminal line dark brown, straight, visible only in posterior two-thirds, embedded with a narrow bright band on its outer side; short black stripes present between each vein on terminal line. Fringe dark brown mixed with black.

**Male genitalia** (Fig. 2a, b). Uncus slender, with a single inward-curved small spine at tip. Tegumen broad, smoothly excurved on lateral margins and slightly concaved on upper margins. Transtilla elbow-shaped, pointed apically. Juxta shield-shaped, slightly sclerotised, membranous on sides in middle and lower parts. Valva narrow, elongated, more or less gradually narrowing towards distal end; costa with a lobe-like process basally, a strongly sclerotised, thorn-shaped process at 3/5 from base and specialised into a large, roughly equilateral triangular-shaped lobe at tip; sacculus slightly enlarged at basal part, then slightly waved, covered with long hairs; cucullus bluntly rounded. Vinculum V-shaped, blunt at distal part. Aedeagus slender, with a near-right angle bend near middle, vesica membranous, long, nearly as long as aedeagus, densely covered with small scobinations; vesica bears two areas of strongly sclerotised conical cornuti arising tightly basally, first one on ventral side formed by a row of short, stout conical cornuti and the other one on dorsal side single, long, large and horn-like.

**Female**. Unknown.

#### Diagnosis

The new species (Fig. 1a, b) is easily distinguishable from its close congeners (Fig. 1c, d, e, f) by external and male genitalia characters: (1) *S.jinggangshana* sp. nov. exhibits an overall grey-white colouration, whereas its close relatives are darker, often sandy-yellow or brownish; (2) the postmedial line of the new species is slender near the costal margin, whereas in *S.takasago* Wu, Fu & Owada, 2013 (Fig. 1c) and *S.cristulalis* (Fig. 1d), it expands into a black spot at the same position; in *S.formosensis* (Fig. 1e), the postmedial line is thinner than that of *S.jinggangshana* and, in *S.japonica* (Fig. 1f), it narrows towards the costal margin; (3) the subterminal line is indistinct in *S.jinggangshana* sp. nov. and *S.japonica*, while it is clear in other species.

In the male genitalia, *S.jinggangshana* sp. nov. (Fig. 2a, b) can be distinguished from close congeners (Fig. 2c, d, e, f) by the following characters: (4) the shape of the proximal margin of the tegumen, which has triangular projections from the proximal corners in *S.jinggangshana* sp. nov., whereas the others lack such projections; (5) the terminal part of the costa with a large, wide triangular projection in the new species, whereas the same structure typically is present in a spiny form in congeners excluding *S.japonica*, which also have a triangular projection, just without the extended round distal margin of the terminal part of the costa like in the new species; (6) the aedeagus is slender, with a nearly right angle bend near the median in *S.jinggangshana* sp. nov., whereas it is shorter, thicker and either not bent or only slightly bent in congeners.

#### Etymology

The specific name is derived from Jinggangshan National Nature Reserve in Jiangxi Province, China, which is the type locality of the new species.

#### Distribution

China: Jiangxi.

#### Biology

The two type specimens were collected in August at altitudes ca. 706 m. The collection site is close to a mixed coniferous and broad-leaved forest, with farmland surrounding it. During the collection period, the area experienced high humidity, frequent rainfall and relatively high temperatures. The immature stages are still unknown.

## Supplementary Material

XML Treatment for Sinarella

XML Treatment for Sinarella jinggangshana

## Figures and Tables

**Figure 1a.**
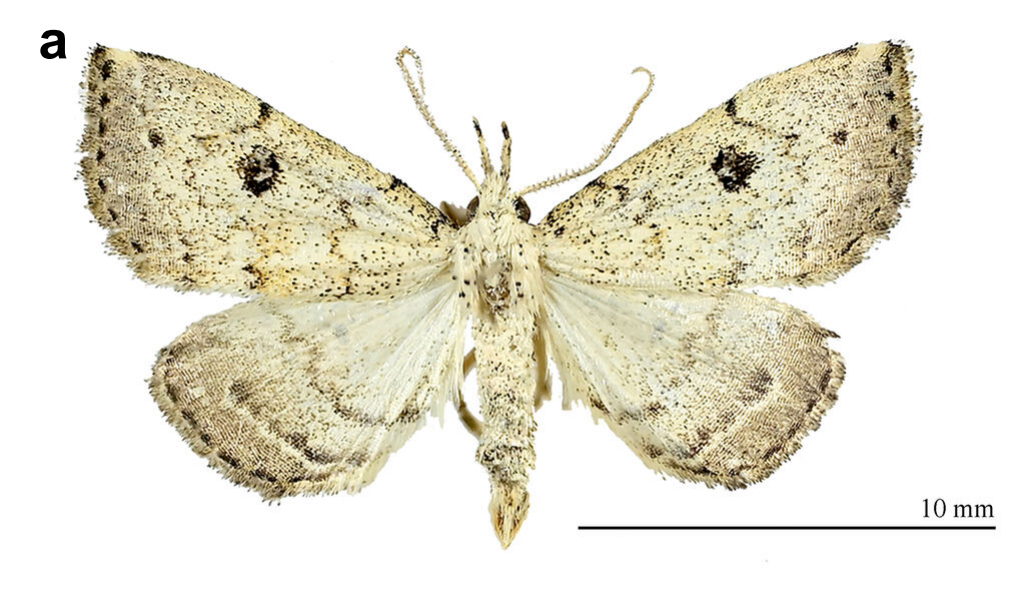
*S.jinggangshana* sp. nov., HT, hhl-6359-1, in NEFU;

**Figure 1b.**
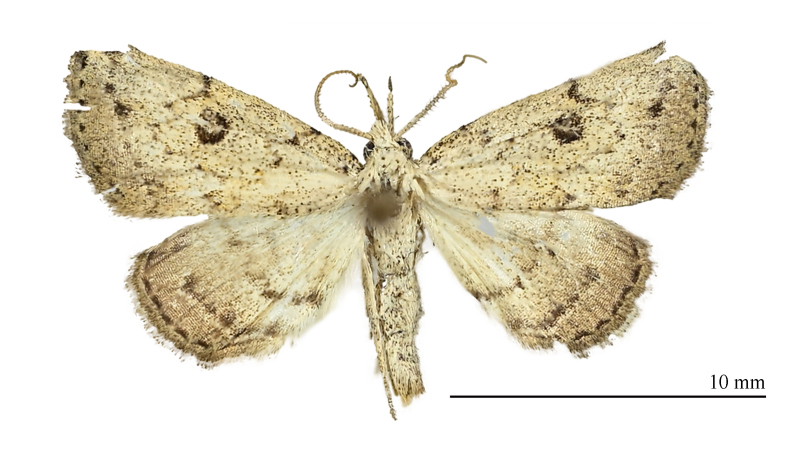
*S.jinggangshana* sp. nov., HT, hhl-6353-1, in NEFU;

**Figure 1c.**
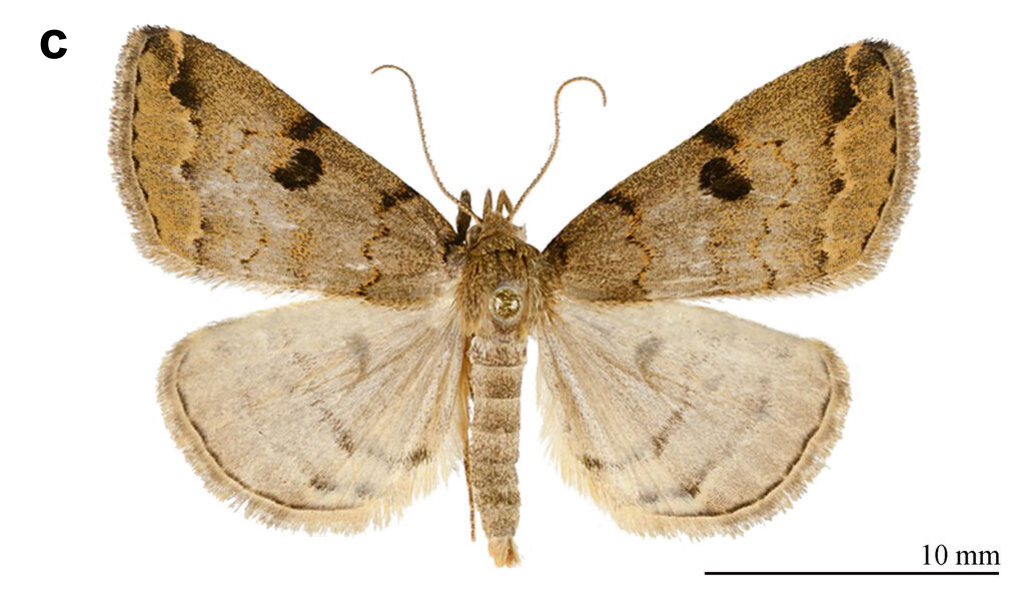
*S.takasago*, HT, photo after Wu (2014);

**Figure 1d.**
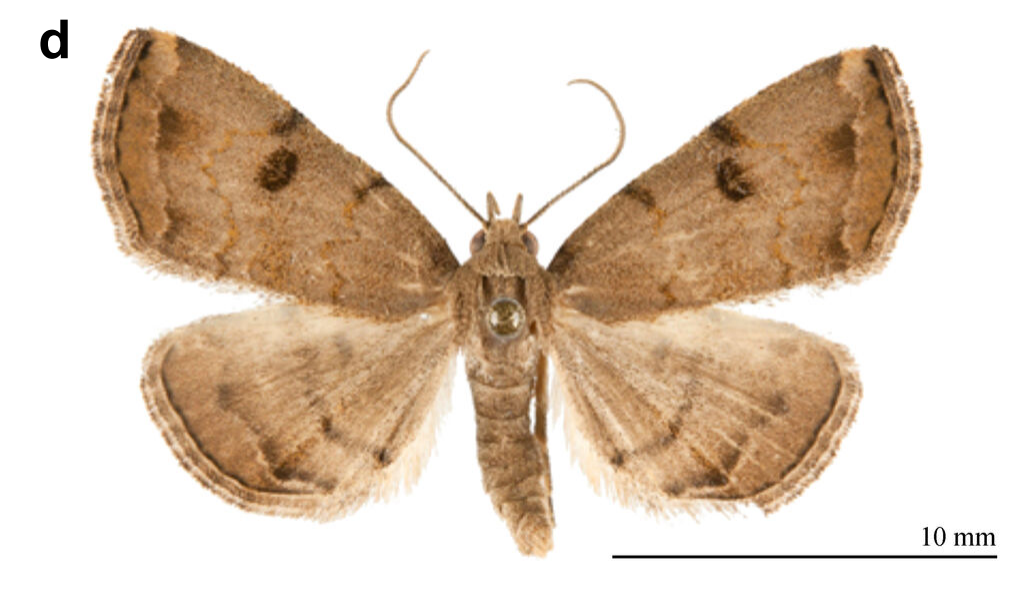
*S.cristulalis*, photo after Wu (2014);

**Figure 1e.**
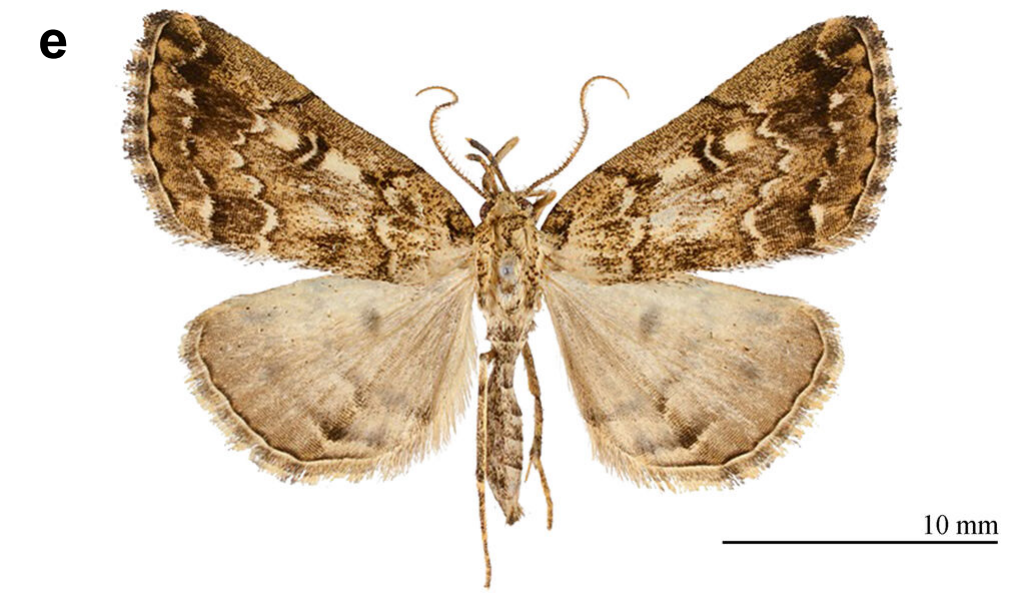
*S.formosensis*, HT, photo after Wu (2014);

**Figure 1f.**
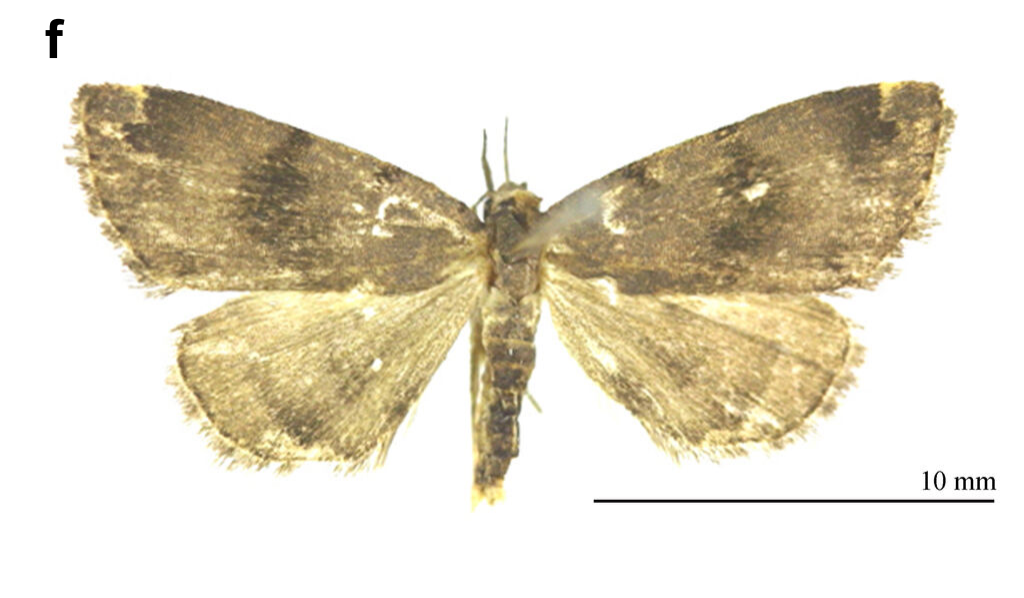
*S.japonica*, in NEFU.

**Figure 2a.**
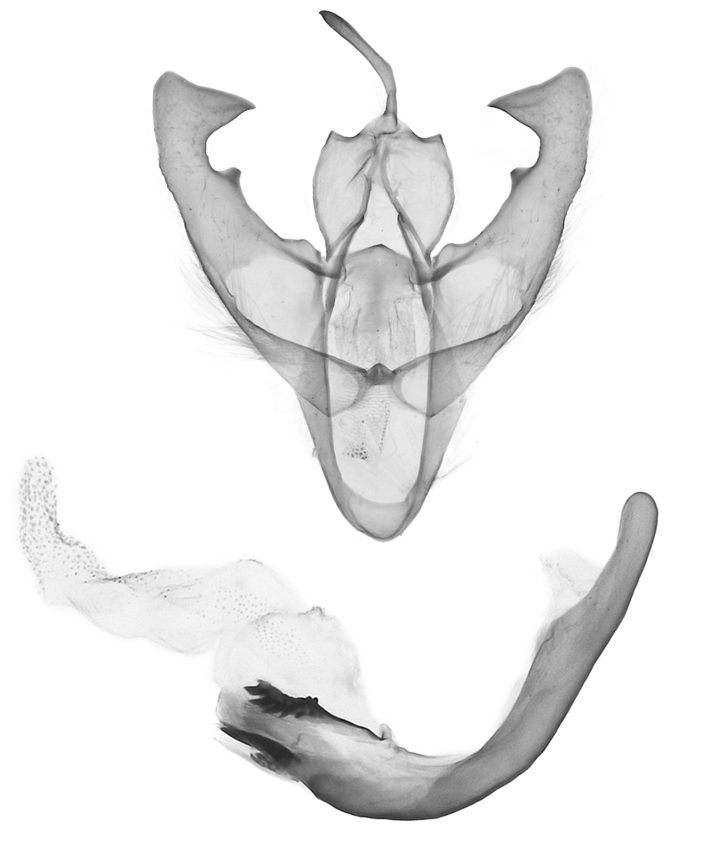
*S.jinggangshana* sp. nov., HT, hhl-6359-1, in NEFU;

**Figure 2b.**
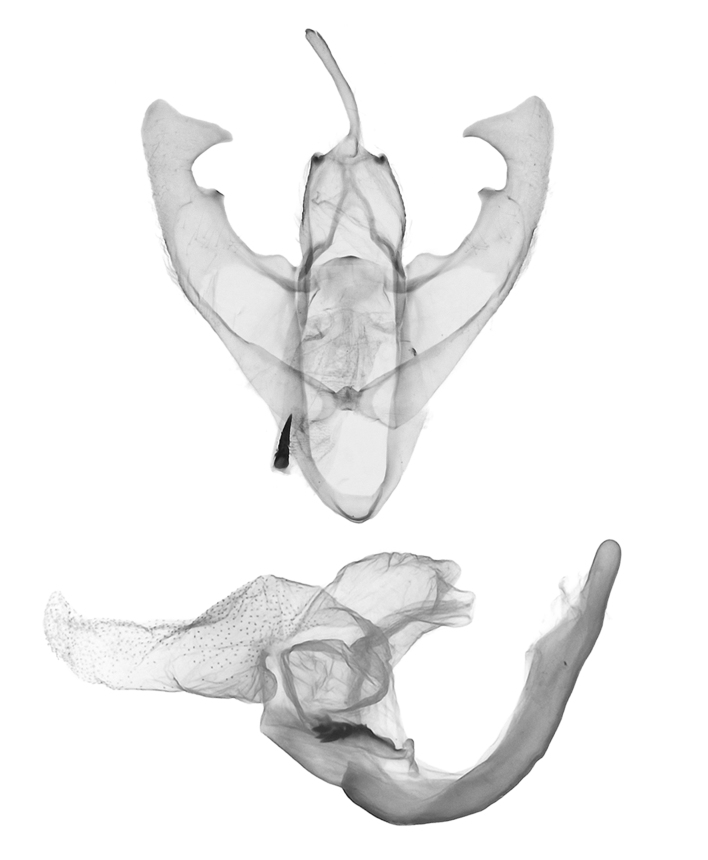
*S.jinggangshana* sp. nov., HT, hhl-6353-1, in NEFU;

**Figure 2c.**
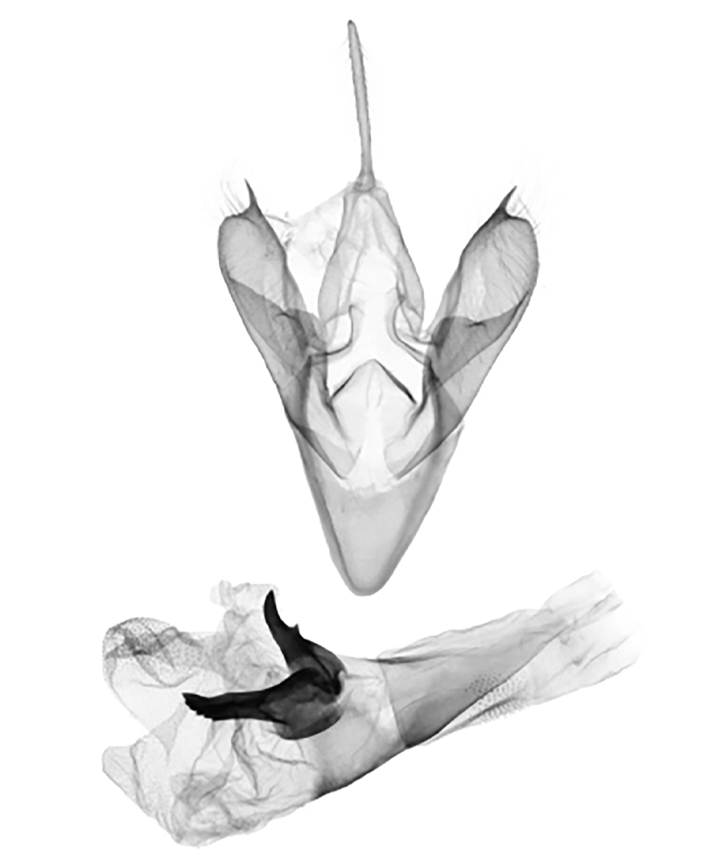
*S.takasago*, HT, photos after Wu (2014);

**Figure 2d.**
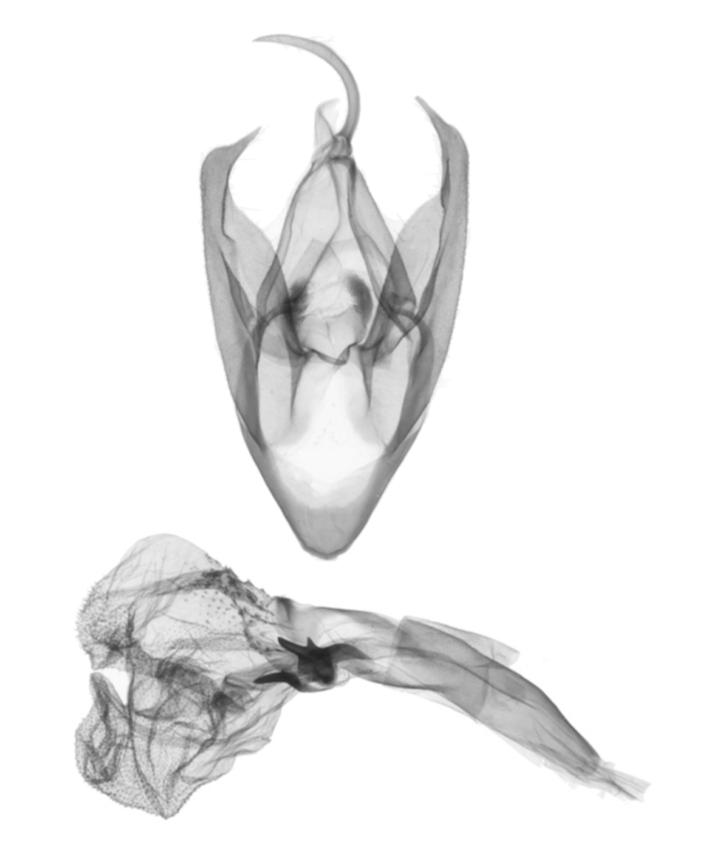
*S.cristulalis*, photos after Wu (2014);

**Figure 2e.**
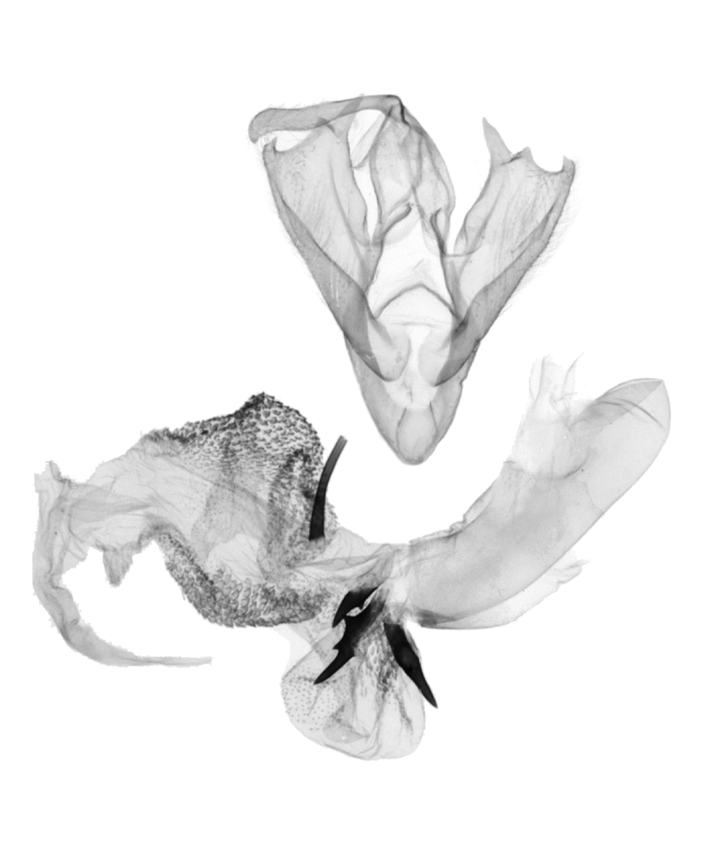
*S.formosensis*, HT, photos after Wu (2014);

**Figure 2f.**
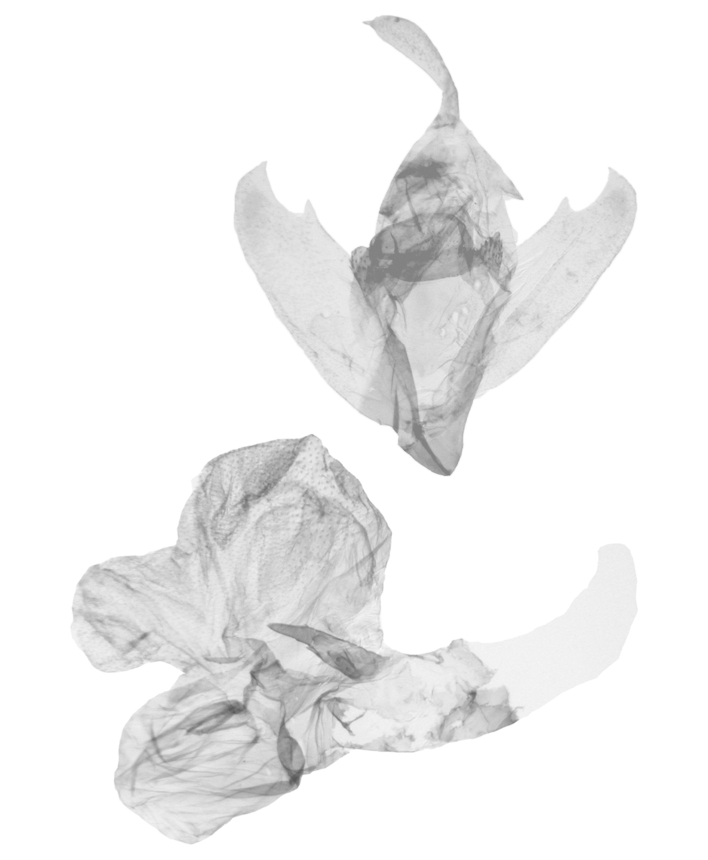
*S.japonica*, photos after Wu (2014).
